# The effects of eccentric hamstring exercise training in young female handball players

**DOI:** 10.1007/s00421-022-04888-5

**Published:** 2022-01-22

**Authors:** Márk Váczi, Gábor Fazekas, Tamás Pilissy, Alexandra Cselkó, Lukasz Trzaskoma, Balázs Sebesi, József Tihanyi

**Affiliations:** 1 Institute of Sport Sciences and Physical Education, University of Pécs, Ifjúság útja 6, 7624 Pécs, Hungary; 2 National Institute for Medical Rehabilitation, Budapest, Hungary; 3 Department of Rehabilitation Medicine, University of Szeged, Szeged, Hungary; 4 Department of Manufacturing Science and Engineering, Faculty of Mechanical Engineering, Budapest University of Technology and Economics, Budapest, Hungary; 5 Doctoral School of Health Sciences, University of Pécs, Pécs, Hungary; 6 Department of Biomechanics, Kinesiology and Informatics, Faculty of Physical Education and Sport Science, Semmelweis University, Budapest, Hungary; 7 Doctoral School of Biology and Sportbiology, University of Pécs, Pécs, Hungary

**Keywords:** Nordic hamstrings, CMJ height, Eccentric training, ACL injury

## Abstract

**Purpose:**

The multidimensional role of hamstring muscle group strength in athletic performance and injury prevention is well documented, and nordic hamstring exercise (NHE) is a popular method for the development of hamstring strength. Our aim was to examine the EMG characteristics of the eccentric NHE as well as the effects of long-term eccentric NHE training on muscle strength and vertical jump performance in 10- to 11-year-old female handball players.

**Methods:**

Players from the same handball team were randomly assigned to an eccentric NHE training (13 players) or a control group (10 players). Both groups continued their regular handball training routine, but the NHE group performed additional eccentric NHE exercises once or twice a week, with progressively increasing volume, over 20 weeks. To test training effects, countermovement jump (CMJ) height, eccentric hamstring impulse, peak torque, and angle of peak torque were evaluated before, during and after the training period. In the pre-exercise test, EMG activity of the medial and lateral hamstring muscle was also assessed during NHE.

**Results:**

Hamstring activities ranged between 98 and 129%. Lateral hamstring activity was greater than medial only in the right leg during NHE. Eccentric hamstring impulse improved in both legs at 10 weeks in both groups. Then, at 20 weeks, it remained unchanged in the NHE but decreased in controls. A similar adaptation was seen in eccentric hamstring torque, without change in the optimum knee angle. CMJ height improved only in the NHE.

**Conclusion:**

It is concluded that NHE activates the hamstring musculature effectively, and a favourable mechanical adaptation to long-term NHE exercise in girls can be triggered as early as 11 years of age.

## Introduction

The multidimensional role of hamstring strength in athletic performance is well understood, and the direct contribution of hamstring strength to athletic skills such as sprinting and jumping has been demonstrated in several investigations (Soylu et al. 2020; Markovic et al. 2020). Hamstring strength also plays an important role in knee joint stabilization, indirectly influencing the qualities of agility manoeuvres like accelerations, decelerations, change of directions, and cuttings (Greig and Naylor 2017; Jones et al. 2017). Moreover, hamstring weakness has been suggested to increase the risks of hamstring strain injury (HSI), a typical sport injury, which incurs in sports with high sprint speed demands and/or concurrent extreme muscle stretch, such as athletics and ball games (Lee et al. 2018).

Because hamstring is a non-antigravity muscle group, its development is limited compared to the antigravity muscles; based on the aforementioned statements, hamstring weakness seems a crucial limitation in athletic preparation. Previous studies have shown that hamstring strength in girls does not increase spontaneously after the age of 11 years, during the adolescent growth spurt (Barber-Westin et al. 2006; Wild et al. 2013), or increase to a lesser extent than the quadriceps strength after the menarche (Ahmad et al. 2006). Also, quadriceps and hamstring strength showed similar development patterns in boys and girls from age 7 to 10, but hamstring strength development lagged behind in girls at age 11. Beside the increased risk of NHI, the hamstring-assisted anterior cruciate ligament (ACL) protection also becomes limited. The low hamstring strength or its insufficient activation is associated with the non-contact ACL injury rates, which is especially high in team games (Zebis et al. 2009). Specifically, the lateral hamstring, as a synergist of the ACL, crosses the knee joint and provides its secondary protection, especially at smaller joint angle. ACL injury risk starts to increase at 12–13 years of age in girls (Granan et al. 2009). The rate of incidence has also been shown to be 1.4-fold greater for girls versus boys (Bram et al. 2021). In addition, reduced hamstring to quadriceps ratio augments the risks for HSI. These findings suggest that hamstring strengthening is necessary in the prevention of ACL injury and HSI in girls, which should start as early as at 11 years of age; and yet, information on the effectiveness of hamstring exercise training is limited to the adult and youth athletic populations (Freeman et al. 2019; Mendiguchia et al. 2020) or boys (Tansel et al. 2008).

Nordic hamstring exercise (NHE) is a popular method for the development of hamstring strength (Chaabene et al. 2020), which is a self-controlled bodyweight resistance variation of the traditional leg-curl exercise. The execution of NHE does not require any equipment and it provides an eccentric stimulus resulting from the weight of the upper body. The favourable adaptive responses in hamstring strength and biceps femoris long head architecture to eccentric hamstring exercise training is well documented (Gérard et al. 2020). High voluntary activation of the hamstring during an eccentric exercise contraction is critical for neural adaptation, and data about the EMG activity of the involved muscles during the NHE are controversial. The lateral part of the hamstring prevents excessive tibial internal rotation and anterior shear force at the knee (Azmi et al. 2018). Some studies using surface EMG found no significant difference between the activity of the medial and lateral hamstring muscles (Iga et al. 2012; Zebis et al. 2013). In contrast, some authors reported preferential activation of the biceps femoris short head (Mendiguchia et al. 2013) or the semitendinosus (Bourne et al. 2016) during the NHE. NHE appears to activate the hamstring in adults effectively (Ebben 2009; Ditroilo et al. 2013), but it remains unknown whether pre-pubertal girls can efficiently activate their hamstring during NHE.

Several studies have shown a significant increase in hamstring strength after a NHE training intervention in adult athletes (Askling et al. 2003; Mjølsnes et al. 2004; Clark et al. 2005; Iga et al. 2012). Despite substantial evidence supporting NHE for young adult populations, limited research has assessed the impact of NHE on strength in pre-pubertal age groups, especially in girls. Closest to the focus of the present work is a study by Tansel et al. (2008), which reported significant increase in eccentric hamstring strength and countermovement jump (CMJ) height following NHE training in 10–12-year-old male basketball players. CMJ height is an important functional measure of athletic performance often studied in NHE interventions (Lindstedt et al. 2002; Clark et al. 2005).

The aim of the present study was two-fold. First, to investigate the EMG characteristics of the medial (semitendinosus, semimembranosus) and lateral (biceps femoris short and long head) hamstring muscles during NHE. Second, to measure the impact of a 20-week NHE training program on hamstring peak torque, impulse, optimum knee angle, and on CMJ height in 10- to 11-year-old female handball players, taking their biological age into account.

## Materials and methods

### Participants

Twenty-three adolescent female players from a national-level handball team were randomly assigned to either a NHE (*n* = 13, age: 11.3 ± 0.5 years, weight = 41.5 ± 7.0 kg; height = 150 ± 6.6 cm) or a control group (*n* = 10, age: 10.9 ± 0.5 years, weight = 41.1 ± 5.6 kg; height = 146.4 ± 3.4 cm). The experiment was approved by the Ethical Committee of the University of Physical Education, Hungary. Participants and their parents were fully informed about the study, subsequently, written informed consent was obtained from them. Players with a history of any lower extremity injury were excluded from the study.

### Experimental protocol

We conducted three test sessions during the 20-week-long intervention study: pre-, mid-, and post-training sessions at 0, 10, and 20 weeke, respectively. In the pre-training test session, beside the anthropometric measurements, hamstring EMG activity during NHE was measured to target our first research purpose. To study the effects of the NHE training intervention, performance measurements (CMJ height, hamstring and quadriceps impulse) were taken at all three test sessions.

During the intervention, both groups continued their regular handball training routine, but the NHE group performed additional eccentric NHE exercises. The regular handball training program was designed as follows: week 1–6 was a pre-season preparation involving fundamental handball technical drills, individual and team tactics, agility, coordination, low-impact plyometric, own-body resistance, and aerobic endurance exercises. Week 7–20 was in-season training involving handball technical and tactical drills, own-body resistance and aerobic endurance exercises as well as games. The number of training sessions was 4–5 per week in both seasons. The NHE intervention started on week 1.

### Electromyography

EMG activity of the medial (semimembranosus, semitendinosus) and lateral part of the hamstring muscles (short and long head of the biceps femoris) were measured during NHE. Participants performed three trials, while verbal encouragement was given to them to control the knee extension speed as long as possible. The same investigator controlled all trials. Trials which did not fulfill the main criteria (detailed below) were excluded from further analysis.

10 mm diameter, circular, bi-polar, silver–silver chloride surface electrodes (SKINTACT FS-50, Robohardware kft, Hungary) were attached to each participant. Prior to attaching the electrodes at each site, the skin was cleaned with alcohol to minimise the impedance of the skin. Electrodes were carefully positioned and secured on the medial and lateral hamstring on both lower extremities according to the SENIAM protocol. The electrode for the medial hamstring was placed midway between the ischial tuberosity and the medial epicondyle of the tibia. The location of the lateral hamstring electrode was midway between the ischial tuberosity and the lateral epicondyle of the tibia. The electrode placement was subsequently checked by manual muscle testing. EMG signals were recorded and amplified by a Zebris CMS70P motion analyser system at a sampling frequency of 600 Hz. Raw EMG signals were processed using MATLAB (version 2011a, MathWorks Inc., Natick, USA). Data were filtered by a 5th order Butterworth filter with cut-off frequencies of 50–300 Hz. Root Mean Square (RMS) was computed with a window of 50 samples and was normalized to the maximal EMG value measured during maximal voluntary isometric contraction (MVIC) of the hamstring. MVIC was measured in a seated position when the hip and knee flexion angle was set at 90°. Participants performed three trials. The trial with the highest RMS value was used for the normalization.

### Estimation of biological age

Biological age is a better predictor of performance than chronological age, and maturation contributes to absolute strength measures (Baechle and Earle 2008); therefore, the prediction and inclusion of biological age in studies using young participants is important. This study adopted morphological age as a valid measure of biological age as proposed and extensively developed by Mészáros and Mohácsi (1983) for the Hungarian population. Morphological age (MA) was calculated by using the following equation:

$${\text{MA = 0}}{.25 } \times {\text{ (HA + BWA + PLA + CA),}}$$

where HA is the age according to height, BWA is the age according to body weight, PLA is the age according to the plastic index, CA is the chronological age and C is the correction factor. The authors developed tables to predict HA, BWA and PLA from height, body weight and the plastic index. The plastic index characterises the development of the bones and muscles and is calculated as the sum of the shoulder width, forearm, and hand circumference. The reliability of the method was checked by the bone age measured with X-ray (*n *= 485). The correlation between morphological age and bone age was high in 11-year-old girls (*r* = 0.92), and the accuracy of the method was ± 0.25 year (Mészáros et al. 2011).

### Dynamometry

The description of the Multicont II dynamometer (Mediagnost, Budapest and Mechatronic Kft, Szeged, Hungary) is detailed elsewhere (Bosco et al. 1995; Rácz et al. 2002; Váczi et al. 2011). Participants completed a standardised warm-up protocol, which included 5 min of cycling on a cycle ergometer at 70 rpm, and 5 min of dynamic stretching of the lower extremity muscles. Participants conductedthree sub-maximal contractions in each test condition to familiarise themselves with the equipment before testing. All testing was performed in a seated position. Thighs were secured with straps to the seat, the lateral epicondyle of the femur was aligned with the centre of rotation of the lever arm, and the hip angle was set at 120°. The range of motion was set at 10°–90°. Four maximal unilateral eccentric contractions were performed with both legs for the hamstring at 60°s^−1^ constant angular velocity and corrected for gravity. There was a 30-s rest between them. Torque–time curves with the highest mechanical impulse were chosen for further analysis. Mechanical impulse (I) was calculated by using the following equation:

$$I = \int\limits_{{t_{1} }}^{{t_{n} }} {M_{(t)} } \cdot {\text{d}}t,$$

where *M* is the torque and *t* is the time. It was chosen as it characterises adaptation in the entire range of motion, while peak torque allows us to detect changes in the optimum angle.

### Countermovement jump height

A contact mat (Chronojump Bosco System, Barcelona) was used to measure flight time and to calculate CMJ height (Blas et al. 2012). CMJ height was determined after the strength measurements following one-hour rest to decrease the effect of fatigue. The participants performed five CMJs after a standardised warm-up (5-min cycling and 5-min dynamic stretching). Participants were asked to place their arms on their waist and maintain that position during the jumps. They executed the jumps with maximal effort using self-selected joint flexion. The best trial was used for statistical analysis.

### NHE training

The NHE group completed a 20-week, progressive NHE training programme besides their regular handball training, two times a week for 10 weeks, then reduced to once per week for an additional 10 weeks (Table 1). The mean participation rate was 93.7%. The NHE sessions were completed after the regular handball training sessions. The control group completed the normal handball training, but did not participate in the NHE sessions.

**Table 1:** Eccentric hamstring training protocol for the NHE group

Week	Sessions per week	Sets and repetitions	Load
1–2	2	2 × 5	–
3	2	2 × 8	–
4	2	2 × 10	–
5–6	2	3 × 8	–
7	2	3 × 10	–
8	2	3 × 12	–
9–10	2	3 × 15	–
11–20	1	3 × 15	Medicine ball (2 kg)

**Fig. 1:**
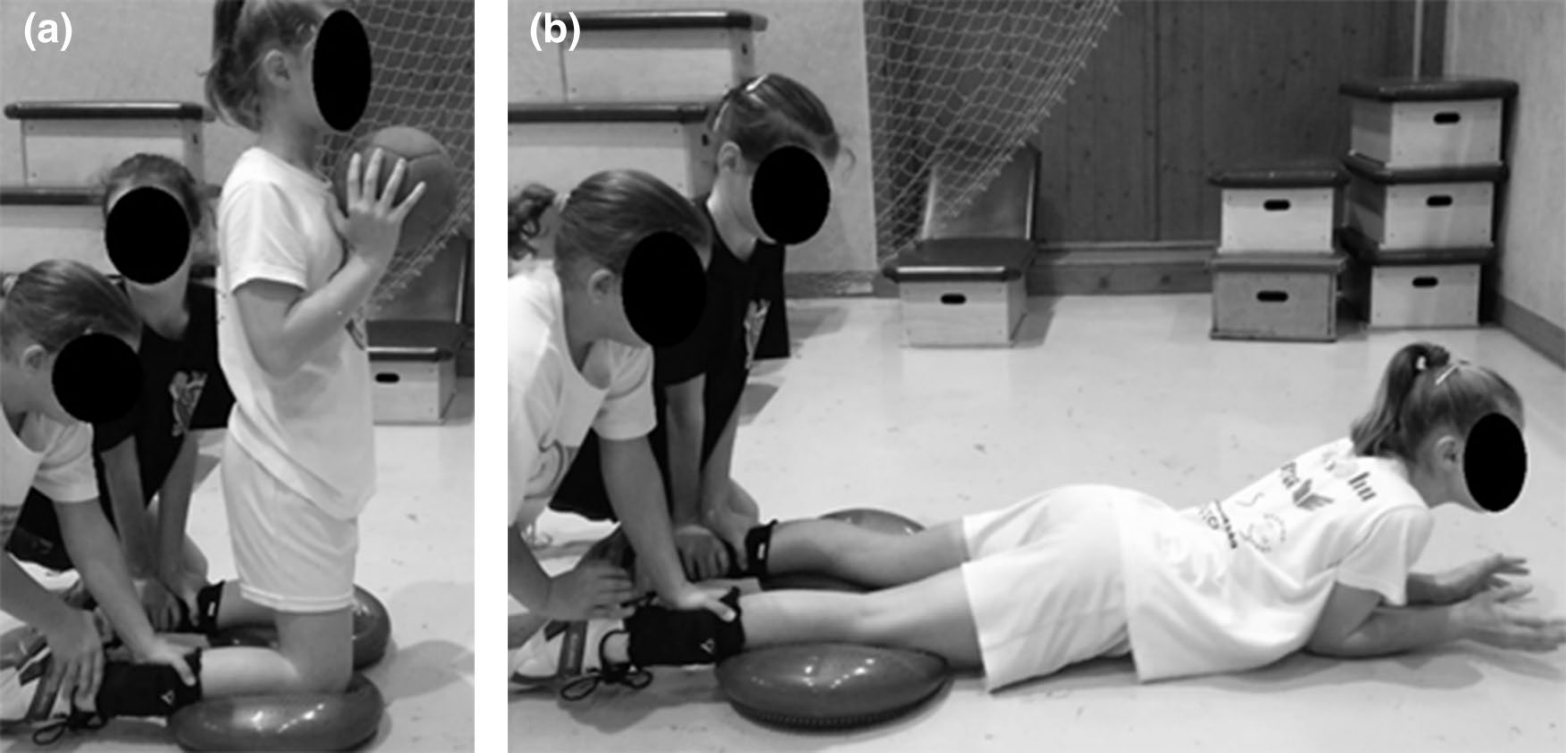
**a** The starting position of the Nordic hamstring exercise. Participants were asked to resist the gravitational force of the torso by the eccentric contraction of the hamstrings. Medicine ball can be used as additional resistance to increase load. **b** The final position of the Nordic hamstring exercise. Before starting the next repetition, participants were asked to push themselves back to the starting position to decrease the concentric work of the hamstrings

NHE is a partner resisted exercise. In groups of three, one participant kneeled in an upright posture whilst her two partners fixed her lower legs above the ankle. Dynair pillows were placed under the knees to decrease the pressure on the knee. The hip joint was in neutral position and the arms were held in front of the chest (a medicine ball was used to increase the load of the hamstring in the later stages of the program). From this starting position the participant tilted forward allowing the moment of the gravitational force of the torso to extend the knee joint (Fig. 1a). The participant controlled the joint extension speed with persistent eccentric contraction of the hamstrings. When a participant was unable to voluntarily resist the moment of the gravitational force, she fell forward and attenuated the impact with her hands (Fig. 1b). Before the next repetition, the participant was required to push herself back to her starting position to decrease the concentric work of the hamstrings. The NHE group executed the eccentric exercise training in the cool down period on days when training volume and/or intensity were low (e.g. days with technical, tactical and/or aerobic endurance exercises).

### Statistics

Data were checked for normality by using the Shapiro Wilk test. We tested peak torque, impulse and CMJ height changes over time, using a two-way mixed design analysis of variance (ANOVA), and testing group (NHE vs. control) by time (pre, 10-wk, 20-wk) interactions. When assumption of sphericity was violated according to the Mauchly’s test, the Greenhouse–Geisser correction was applied. In case of interaction, a one-way analysis of variance (ANOVA) with repeated measures was used to compare group means. When significant group main effect was found, independent *t*-tests and Hedge’s *g* effect sizes were applied to determine the differences between the groups at Pre, 10-wk, and 20-wk time points. The magnitude of the effect size (ES) was classified as trivial (< 0.2), small (> 0.2–0.6), moderate (> 0.6–1.2), large (> 1.2–2.0) and very large (> 2.0–4.0) based on guidelines from Batterham and Hopkins (2006). To determine differences in EMG activity between the medial and lateral hamstring muscles, and biological age between groups, independent t-tests were used. The level of significance was set at 0.05.

## Results

### EMG data

The mean RMS (normalized to MVIC) values were as follows: left medial hamstring = 107.56 ± 30.52%, left lateral hamstring = 128.99 ± 10.16%, right medial hamstring = 98.83 ± 28.89%, right lateral hamstring = 118.41 ± 46.99%. The left medial hamstring activity was significantly higher than the right medial hamstring activity (*t* = − 2.82; *P* = 0.007). There was no inter-limb difference in lateral hamstring activity. In the right leg, lateral hamstring activity was greater than the medial (*t* = 2.56; *P* = 0.014). The same tendency was seen in the left leg, but the difference was not significant (*t* = 1.47; *P* = 0.15).

### Biological age

The estimated morphological age was 11.7 ± 0.8 and 11.5 ± 0.6 years in the NHE and in the control group, respectively; the difference between the groups was not significant (*t* = 0.483; *P* = 0.64).

### Eccentric hamstring impulse

**Fig. 2:**
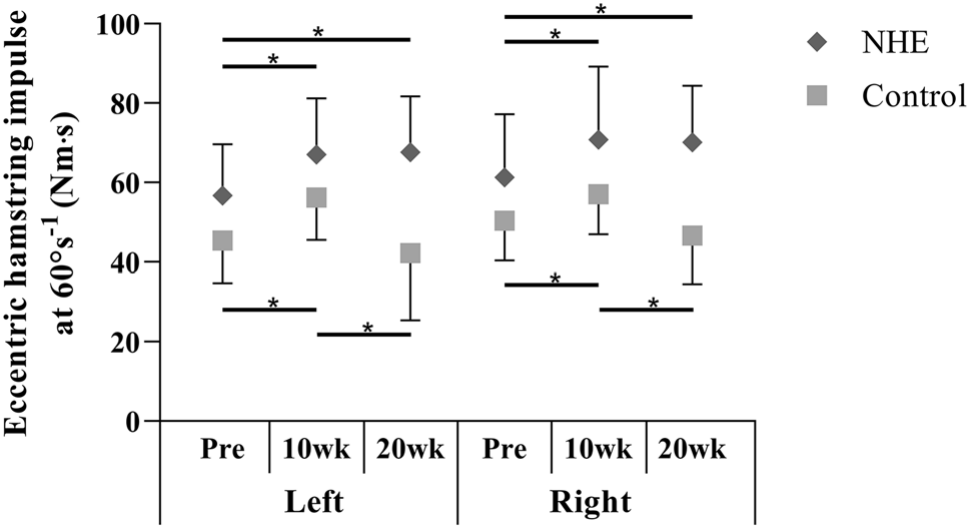
Mean (± SD) eccentric hamstring impulse at 60°s^−1^ constant angular velocity before the training intervention (Pre), after 10 (10 wk) and 20 weeks (20 wk) in the NHE and the control group. * Significant difference (*p* ≤ 0.05)

There was no significant difference between groups in eccentric hamstring impulse for any legs at Pre. Significant group by time interaction was found for the eccentric hamstring impulse in the left limb (*F*_2,42_ = 5.20; *P* = 0.010). In the NHE group the hamstring impulse in the left leg was significantly greater at 10 wk (*P* = 0.030) and at 20 wk (*P* = 0.002) than at Pre. In the control group the left eccentric hamstring impulse was significantly greater at 10 wk than at Pre (*P* = 0.010), and at 20 wk it was significantly less than at 10 wk (*P* = 0.000) (Fig. 2).

A significant group by time interaction was also revealed for the eccentric hamstring impulse in the right limb (*F*_2,42_ = 4.89; *P* = 0.01). In the NHE group, hamstring impulse in the right leg was significantly greater at 10 wk (*P* = 0.02) and at 20 wk (*P* = 0.002) than at Pre. In the control group, the right eccentric hamstring impulse was significantly greater at 10 wk than at Pre (*P* = 0.017), and at 20 wk it was significantly less than at 10 wk (*P* = 0.004) (Fig. 2).

### Eccentric hamstring peak torque

**Fig. 3:**
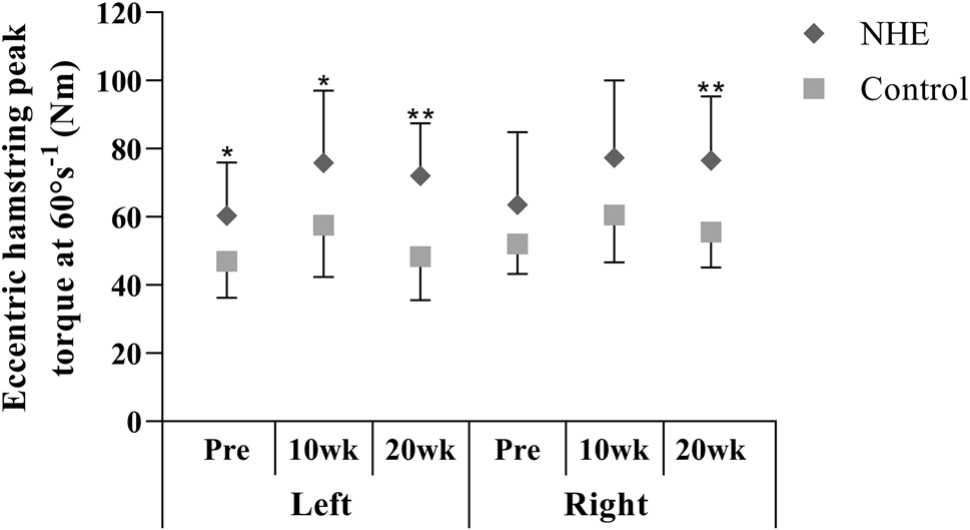
Mean (± SD) eccentric hamstring peak torque at 60°s^−1^ constant angular velocity before the training intervention (Pre), after 10 (10 wk) and 20 weeks (20 wk) in the NHE and the control group. *Significant difference (*p* ≤ 0.05); **significant difference (*p* ≤ 0.01) between the groups

A significant group main effect was found for the eccentric hamstring peak torque of the left (*F*_1,21_ = 10.51; *P* = 0.004) and the right limb (*F*_1,21_ = 15.53; *P* = 0.01). The difference between the NHE and control group in the left leg at 20 wk (*t* = 4.0; *P* = 0.001; ES = 1.68) was greater than at Pre (*t* = 2.35; *P* = 0.029; ES = 0.98). The difference between the NHE and control group in the right leg was not significant at Pre (*t* = 1.59; *P* = 0.13, ES = 0.63), but increased at 20 weeksk (*t* = 3.46; *P* = 0.002; ES = 1.46) (Fig. 3).

### Optimum knee angle

**Fig. 4:**
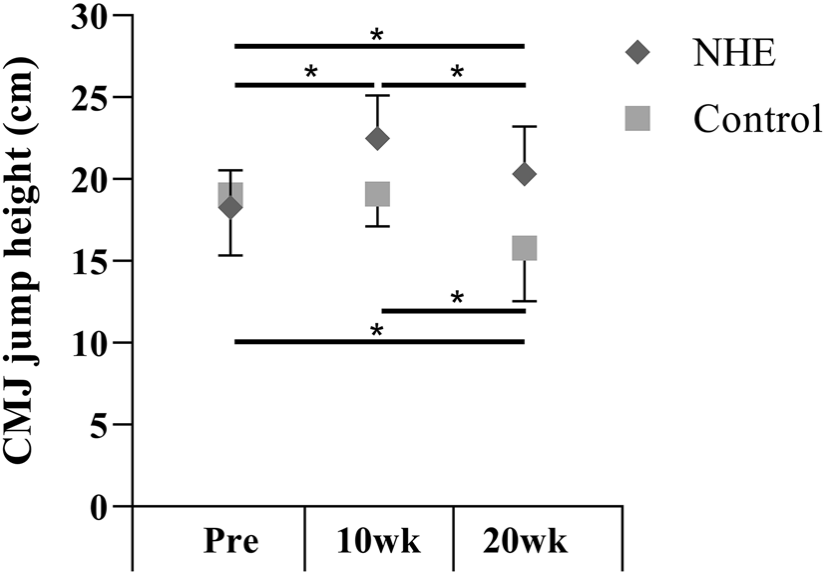
Mean (± SD) optimum knee angle obtained during eccentric hamstring contraction at 60°s^−1^ constant angular velocity before the training intervention (Pre), after 10 (10 wk) and 20 weeks (20 wk) in the NHE and the control group

There was neither significant group by time interaction nor significant main effects in optimum knee angle obtained during eccentric hamstring contraction (Fig. 4).

### CMJ height

**Fig. 5:**
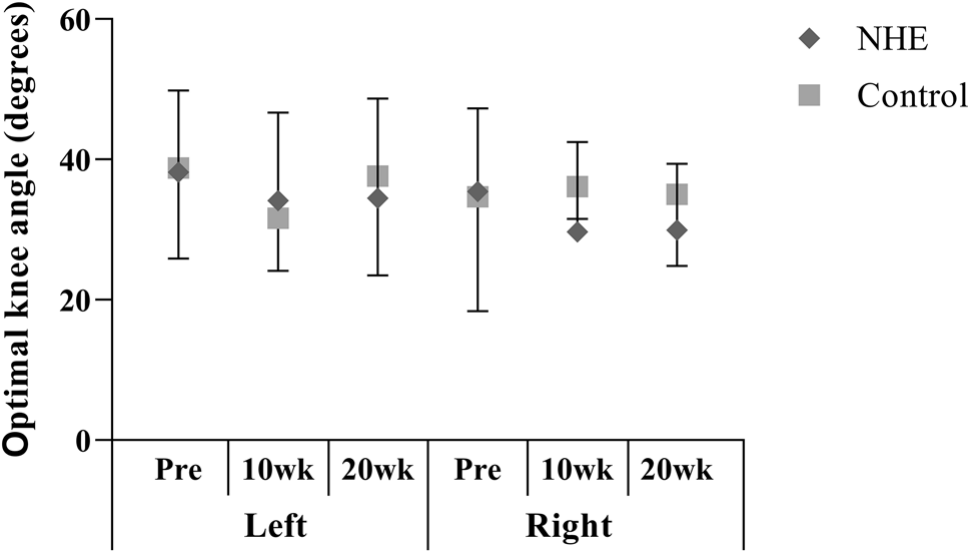
Mean (± SD) countermovement jump height before (Pre) the intervention, after 10 (10 wk) and 20 weeks (20 wk) in the NHE and control group. *Significant difference (*p* ≤ 0.05)

A significant group by time interaction was found in the CMJ height (*F*_1.554,32.64_ = 10.57; *P* = 0.001). In the NHE group, CMJ height was significantly greater at 20 wk (*P* = 0.038) and at 10 wk (*P* = 0.000) than at Pre; at 10 wk it was significantly greater than at 20 wk (*P* = 0.038). In the control group, CMJ height was significantly less at 20 wk than at 10 wk (*P* = 0.03) and at Pre (*P* = 0.003) (Fig. 5).

## Discussion

In the present study, our EMG data show that NHE activated the hamstrings effectively; the lateral part of the hamstring was affected at a greater extent in 10- to 11-year-old female handball players. The 20-week-long eccentric hamstring strengthening program induced favourable changes in hamstring eccentric peak torque and impulse, without any change in the optimum knee angle. Furthermore, the strength changes accumulated into functional improvement, as shown by the increased CMJ height in the NHE group.

Our participants activated the hamstring muscles effectively (between 98 and 129% of MVIC) during NHE. Biceps femoris activation was greater than the semitendinosus in the right leg. The trend was the same in the left leg, but the difference failed to reach significance. In contrast, previous studies found no difference between the medial and lateral part of the hamstring (Iga et al. 2012; Zebis et al. 2013). Using the T2 of the MRI, Mendiguchia et al. (2013) and Bourne et al. (2013) came to contradicting conclusions. After 5 × 8 repetitions of NHE, the short head of the biceps femoris close to the origin was loaded the most (Mendiguchia et al. 2013). However, after 6 × 10 repetitions of NHE, the percentage increase in the T2 was highest in the semitendinosus (Bourne et al. 2016). Direct comparison of the results is difficult, as the number of sets and repetitions could influence the results. Marshall et al. (2015) showed that the medial hamstring EMG activity increased from the first set; however, those of the biceps femoris only increased from the third set during the descent phase of the NHE. In contrast, the medial hamstring activity did not change, but the biceps femoris EMG decreased in the ascent phase from the first set of 6 × 5 repetitions of NHE. These results suggest that the medial and lateral part of the hamstring might respond differently to several sets of NHE, which might explain the discrepancies within the literature concerning the EMG activity pattern of the NHE. In addition, possibly gender differences should be taken into consideration, as most of the aforementioned studies measured young adult males in comparison to the youth female participants in our study (Ebben 2009).

Strength development in young players is often associated with biological age (Mészáros et al. 2000). However, the previous study investigating the effects of NHE on male basketball players of a similar age failed to control this important factor (Tansel et al. 2008). In the present study, there was no difference in the estimated biological age between groups, suggesting that the observed changes in the mechanical and CMJ variables were caused by the training intervention and not by the different maturational status of the adolescent girls in the NHE and the control group.

The lack of group by time interaction in the present study suggests that eccentric hamstring peak torque increased similarly in the NHE group and in the controls after 10 weeks in both legs. The multimodal characteristics of general handball training may explain this finding. According to meta-analyses, multimodal activities including strength, balance, power and agility exercises reduce injury rate and increase athletic performance (Faude et al. 2017; Vatovec et al. 2020). In our study, for example, the plyometric exercises themselves, applied in the first 6 weeks of the normal handball training program as part of the general preparation phase, may have enhanced the eccentric hamstrings peak torque (Hewett et al. 1996). We observed remarkable improvement in the eccentric hamstring peak torque in the NHE (+ 22–25%) and the control group (+ 15–23%) after 10 weeks. However, after 20 weeks, the values in the NHE groups were still markedly higher than before the start of the intervention (+ 20%), while the values returned to the pre-training level in the control group (+ 4–5.7%). Although there was a difference in eccentric hamstring strength between groups at Pre (ES = 0.98) in the left leg, the difference was greater at 20 wk (ES = 1.68). There was no difference in eccentric hamstring strength between groups at Pre in comparison to 20 wk (ES = 1.46) in the right limb. These findings suggest that NHE training was effective in increasing eccentric hamstrings strength, and regular handball training might not be sufficient to maintain the strength gains elicited by its multimodal training stimuli during the preparation phase. We believe that the eccentric hamstring impulse is a better marker of the strength training adaptation than the peak torque, as it characterises the adaptation in the whole range of motion. The group by time interaction for both legs in these variables suggests that, although general preparation efficiently increased eccentric hamstring impulse in the first 10 weeks, handball training alone was not sufficient to maintain these benefits. Therefore, NHE seems to be efficient at increasing and maintaining eccentric hamstring impulse in young female handball players when applying 1–2 times weekly.

Earlier investigations demonstrated that NHE programs of 4–10 weeks of duration improved eccentric hamstring strength by 11–21% in adult male soccer players (Mjølsnes et al. 2004; Iga et al. 2012) and in young male basketball players (Tansel et al. 2008). In contrast, Sato et al. (2011) reported that a 6-week (3 × week) program did not increase the eccentric hamstring peak torque in adult females (+ 8.8%), in contrast with males who demonstrated a 20% improvement. The authors suggested that caution is needed when NHE is instructed to women. Although we did not measure muscle damage and soreness with questionnaires (Mjølsnes et al. 2004) or creatine-kinase levels (Váczi et al. 2011), the subjective feedback from the participants showed that two training sessions a week were a sufficient stimulus. Alternatively, the relatively short (6 weeks) training period or the small sample size (8 women) might explain why Sato et al. (2011) did not find any improvement in the eccentric hamstring peak torque of females.

Previous studies have shown that hamstring strength in girls does not increase after the age of 11 years (Barber-Westin et al. 2006) and during the adolescent growth spurt (Wild et al. 2013), or it increases to a lesser extent than the quadriceps strength after the menarche (Ahmad et al. 2006). Therefore, the findings of our study have important clinical relevance in HSI prevention and ACL protection. As ACL injury risk starts to increase at 12–13 years of age in girls (Granan et al. 2009), and hamstring strengthening is one important part of the successful ACL prevention programs (Sugimoto et al. 2015), hamstring strengthening might be advisable in young females before the adolescent growth spurt. Efficient activation of the lateral hamstring has been suggested to reduce the tibial internal rotation torque and the anterior shear force at the knee (Azmi et al. 2018). Our data suggest that the NHE preferentially activated the lateral part of the hamstring (129% and 118% in left and right, respectively), providing important training stimulus to target ACL protection. Hamstring strength and optimum knee angle are also considered promising measures of HSI and ACL injury risk. The hamstring’s susceptibility to eccentric damage as well as ACL injury risk is high, for example, if hamstring peak torque occurs at greater knee joint (Proske et al. 2004; Hiemstra et al. 2004). Though our NHE group demonstrated favourable adaptation in hamstring strength, the NHE program failed to reduce the optimum knee angle. It is possible that children underload their hamstring to avoid excessive tension when the knee is about to extend, yet eccentric training at longer muscle length is preferential for evoking favourable mechanical and morphometric adaptations (Marušič et al. 2020).

The transfer of strength adaptation to functional performance after eccentric hamstring training is in the interest of strength specialists and coaches. Fully supporting our hypothesis, we found an improved CMJ height in the NHE group after 10 weeks (19%), but no changes were found in the control group, suggesting that the strength gain was only transferable to functional performance in the NHE group. These findings are in agreement with those of Tansel et al. (2008), who reported a 10% increase in jump height after a 5-week-NHE intervention in 10- to 12-year-old male basketball players. Anastasi and Hamzeh (2011) reported a 16% increase in CMJ height after a 10-week NHE intervention in female rugby union players. Clark et al. (2005) reported a 7% increase in vertical jump height (4-week NHE intervention), but interestingly, in their study the quadriceps peak torque decreased (11%). The authors suggested that the shift in optimum angle for concentric hamstring torque caused the increased jump height. Specifically, the knee stability may have increased during take-off and the force production was, therefore, more efficient. Although there was no change in the optimum angle for eccentric hamstring toque between 10 and 20 wk and the eccentric hamstring peak torque remained unchanged, the CMJ height in the NHE group decreased from 10 to 20 wk. At 20 wk, however, it was still significantly higher than at Pre. These results suggest that the training volume had an important effect on CMJ height, as the participants executed two eccentric trainings weekly in the first training period, which decreased to one in the second training period. It seems that decreasing the training frequency (only one NHE session per week) during the second 10 weeks was not a sufficient stimulus to further increase the reactive strength during CMJ. Further studies are needed to explore the mechanism of CMJ height improvements after eccentric hamstring training (quadriceps peak torque, optimum angle for concentric hamstring toque, eccentric hamstrings torque). In addition, the decreased CMJ height in the control group suggests that regular handball training alone might not be sufficient to maintain the improvement in jumping ability, in contrast with the preparation period.

An important limitation of this study is the lack of hip angle control during the training intervention. Though all NHE executions were supervised and instructions were given to extend the hip, this may directly influence the proximal/distal activation of the hamstring group and, therefore, can account for changes in the intervention. Another limitation is that the length and the weight of the torso influence knee joint moment during NHE. Therefore, the force needed to control NHE movment could be variable across participants, leading to different training effects on the hamstring. Finally, though hamstring activation was high during NHE in our players, the optimum knee angle was unchanged after the NHE program. Therefore, the EMG-knee angle-time characteristics of the NHE exercise itself should be studied to clarify whether young athletes are able to activate their hamstring in the end-phase of the NHE exercise.

## Conclusion

According to the EMG data in our study, the NHE activated more the lateral part of the hamstring, providing important training stimulus for ACL protection. We also demonstrated that a 20-week-long NHE training increased hamstring torque and impulse without changing the optimum knee angle for hamstring torque. NHE should be incorporated into regular training to increase eccentric hamstring strength in 10- to 11-year-old female handball players, though specific eccentric hamstring exercises are warranted to target hamstring strength improvements at longer muscle length. The pieces of information learned from the present study have important relevance in the prevention of HSI and ACL rupture and in the development of functional performance.

## Data Availability

Not applicable.

## Abbreviations

ACL: Anterior cruciate ligament
BWA: Age according to body weight
C: Correction factor
CA: Chronological age
CMJ: Countermovement jump
CO: Control group
EMG: Electromyography
ES: Effect size
HA: Age according to height
I: Mechanical impulse
M: Torque
MA: Morphological age
MVIC: Maximal voluntary isometric contraction
NHE: Nordic hamstring exercise
PLA: Age based to plastic index
RMS: Root Mean Square
SENIAM: Surface EMG for non-invasive assessment of muscles
*t*: Time
wk: Week
